# Cytochrome P450 monooxygenase of *Acanthamoeba castellanii* participates in resistance to polyhexamethylene biguanide treatment

**DOI:** 10.1051/parasite/2021074

**Published:** 2021-11-10

**Authors:** Jian-Ming Huang, Pin-Ju Ko, Chao-Li Huang, Po-Wei Wen, Chun-Hsien Chen, Min-Hsiu Shih, Wei-Chen Lin, Fu-Chin Huang

**Affiliations:** 1 Department of Parasitology, College of Medicine, National Cheng Kung University Tainan 701 Taiwan; 2 Department of Microbiology and Immunology, College of Medicine, National Cheng Kung University Tainan 701 Taiwan; 3 Institute of Tropical Plant Sciences and Microbiology, National Cheng Kung University Tainan 701 Taiwan; 4 School of Medicine, College of Medicine, National Cheng Kung University Tainan 701 Taiwan; 5 Institute of Basic Medical Sciences, College of Medicine, National Cheng Kung University Tainan 701 Taiwan; 6 Department of Ophthalmology, National Cheng Kung University Hospital Tainan 701 Taiwan

**Keywords:** *Acanthamoeba*, Polyhexamethylene biguanide, P450 monooxygenase, Amoebic keratitis

## Abstract

*Acanthamoeba* spp. are free-living parasites that can cause severe infections such as granulomatous amoebic encephalitis (GAE) and amoebic keratitis (AK). Polyhexamethylene biguanide (PHMB) is a topical application for AK treatment. However, PHMB is not entirely effective against all *Acanthamoeba* strains or isolates. The mechanisms by which *Acanthamoeba* protects itself against extreme drug conditions without encystation are still unknown. According to a previous study, cytochrome P450 monooxygenase (CYP450MO) plays an important role in the oxidative biotransformation of numerous drugs related to metabolism. In this study, a CYP450MO fragment was inserted into the pGAPDH-EGFP vector and transfected into *Acanthamoeba castellanii*. We found that CYP450MO-overexpressing *Acanthamoeba* had higher survival rates than those of the control cells after PHMB treatment. Moreover, we also found that encystation-related genes such as cellulose synthase I (CSI), encystation-mediating serine proteinase (EMSP), and autophagy-related protein 8 (ATG8) expression levels were not significantly different between *Acanthamoeba* transfected by pGAPDH-EGFP or pGAPDH-EGFP-CYP450MO. We suggest that *Acanthamoeba* transfected by pGAPDH-EGFP-CYP450MO may not induce encystation-related genes to resist PHMB treatment. In conclusion, these findings indicate that CYP450MO may be an additional target when PHMB is used for treatment of amoebic keratitis.

## Abstract (fr)

Les *Acanthamoeba* spp. sont des parasites libres qui peuvent provoquer des infections graves telles que l’encéphalite amibienne granulomateuse (EAG) et la kératite amibienne (KA). Le polyhexaméthylène biguanide (PHMB) est une application topique pour le traitement de la KA. Cependant, le PHMB n’est pas entièrement efficace contre toutes les souches ou isolats d’*Acanthamoeba*. Les mécanismes par lesquels *Acanthamoeba* se protège contre des conditions médicamenteuses extrêmes sans enkystation sont encore inconnus. Selon une étude précédente, la monooxygénase du cytochrome P450 (CYP450MO) joue un rôle important dans la biotransformation oxydative de nombreux médicaments liés au métabolisme. Dans cette étude, un fragment CYP450MO a été inséré dans le vecteur pGAPDH-EGFP et transfecté dans *Acanthamoeba castellanii*. Nous avons constaté que les *Acanthamoeba* surexprimant le CYP450MO avaient des taux de survie plus élevés que ceux des cellules témoins après un traitement au PHMB. De plus, nous avons également constaté que les gènes liés aux enkystations tels que la cellulose synthase I (CSI), la sérine protéinase médiatrice de l’enkystation (EMSP) et les niveaux d’expression de la protéine 8 liée à l’autophagie (ATG8) n’étaient pas significativement différents entre les *Acanthamoeba* transfectés par pGAPDH-EGFP ou par pGAPDH-EGFP-CYP450MO. Nous suggérons que les *Acanthamoeba* transfectés par pGAPDH-EGFP-CYP450MO ne peuvent pas induire les gènes liés à l’enkystation pour résister au traitement PHMB. En conclusion, ces résultats peuvent indiquer que la monooxygénase du cytochrome P450 peut être une cible potentielle pour le traitement par PHMB de la kératite amibienne.

## Introduction

*Acanthamoeba* spp. are free-living pathogenic protozoa that are distributed in several environments, including swimming lakes, pools, soil, and dust [6]. *Acanthamoeba* spp. cause severe sight-threatening infections such as granulomatous amoebic encephalitis (GAE) and amoebic keratitis (AK) [25, 37]. AK has been increasing with contact lens misuse over the past two decades [1, 4, 6, 7]. *Acanthamoeba* infects patients by causing lid edema, photophobia, epithelial defects, and ring-like stromal infiltrates through injury to the cornea [20, 24]. Patients with AK have been treated effectively over the last two decades with topical biguanides; however, current therapy requires surgical intervention because of the failure of medical treatment [15]. Polyhexamethylene biguanide (PHMB) is a polymeric biguanide used as a disinfectant and antiseptic for patients with AK [19, 22]. PHMB is effective against *Pseudomonas aeruginosa*, *Staphylococcus aureus*, *Escherichia coli*, *Candida albicans*, and *Aspergillus brasiliensis* [2, 13, 26, 38, 39]. PHMB contains highly charged positive molecules that bind to the phospholipid bilayer of the cell membrane, which is negatively charged, causing penetration, damage, cell lysis, and death of the pathogens [21]. A previous study showed that 0.01% PHMB could not induce obvious corneal toxicity but lysed *Acanthamoeba* after treatment *in vitro* [10, 22]. Combined AK treatment with propamidine, neomycin, and PHMB reduced pain in all patients within 2–4 weeks [36]. PHMB combined with H_2_O_2_ is also used as an ingredient in contact lens-cleaning solutions to prevent corneal infections [30]. Corneal transplantation is another therapeutic approach when topical treatment fails. Nevertheless, corneal transplantation does not eliminate all trophozoites or cysts that can grow in the new cornea. Hence, there are no clinical therapeutic approaches recommended for incorporation into standard practice.

Cytochrome P450 enzymes (CYP450s) involved in drug metabolism are widely identified in different organisms ranging from protozoa to mammals [9, 32, 40]. CYP450s bind and activate two atoms of oxygen from substrates such as peroxide, and lead to hydroxylation [3]. CYP450s also depend on monooxygenase activity, catalyzing the oxidation of endogenous and exogenous substrates, and thereby cause drug degradation [35]. The metabolism of drugs by CYP450s contributes to the formation of products that are less toxic and are excreted easily into cells. *Plasmodium berghei* and *Plasmodium falciparum* can induce CYP450s to exhibit resistance to chloroquine treatment [28]. However, clinical isolates of *Acanthamoeba* with high resistance to PHMB are associated with serious health consequences in Taiwan [10]. Therefore, cytochrome P450 monooxygenase (CYP450MO) may play an important role in the oxidative biotransformation of numerous drugs during drug metabolism in *Acanthamoeba*. In this study, we overexpressed CYP450MO in *Acanthamoeba* to investigate its effects. CYP450MO-overexpressing *Acanthamoeba* had higher survival rates than those of the control cells after PHMB treatment. We suggest that CYP450MO in *Acanthamoeba* may catalyze PHMB drug metabolism to enhance survival rates after PHMB treatment. In conclusion, these findings may help to develop potential treatments for AK patients.

## Materials and methods

### *Acanthamoeba castellanii* cultivation

Trophozoites of *A. castellanii* (Neff strain, ATCC No. 30010, Pacific Grove, CA, USA) were axenically cultured at 28 °C in peptone-yeast extract-glucose (PYG) medium (20 g/L proteose peptone, 2 g/L yeast extract, 0.1 M glucose, 4 mM MgSO_4_, 3.4 mM sodium citrate, 0.9 mM Fe (NH_4_)_2_(SO_4_)_2_, 1.3 mM Na_2_HPO_4_, and 2 mM K_2_HPO_4_, pH 6.5) in cell culture flasks.

### Total RNA isolation and cDNA synthesis

A total RNA Extraction Miniprep System (Viogene, Taiwan) was used to isolate RNA. The total concentration and A260/A280 ratio of mRNA were measured using ND-1000 (NanoDrop, Thermo Fisher Scientific, USA). High-capacity cDNA Reverse Transcription kits (Thermo Fisher Scientific) were used in this study. The reverse transcription conditions were set at the following times and temperatures: 25 °C for 10 min, 37 °C for 120 min, and 85 °C for 5 min; finally, the cDNA was kept at 4 °C. The reaction volume was 20 μL.

### Polymerase chain reaction (PCR)

PCR products were separated on a DNA VIEW (BIOTOOLS Co., Ltd.) – stained gel via agarose gel electrophoresis. The 18S rDNA forward primer F900 was 5′ – CCC AGA TCG TTT ACC GTG AA – 3′, and the reverse primer R1100 was 5′ – TAA ATA TTA ATG CCC CCA ACT ATC C – 3′, which produced 180-bp amplification bands. CSI forward primer was 5′ – GGC GAA GAA CAC CTG GTT AC – 3′, and the reverse primer was 5′ – TGC TCT ACA ACA CGG AGG TG – 3′, which produced 239-bp amplification bands. ATG8 forward primer was 5′ – AAG GAA GCA CAT GAA GCT GAG C – 3′, and the reverse primer was 5′ – CCA TCC TCG TCC TTG TAC TTG G – 3′, which produced 117-bp amplification bands. EMSP forward primer was 5′ – CAA CTA CAC CCA GGA CAC CC – 3′, and the reverse primer was 5′ – GGT CTA CAA AGC GGG AGA GG – 3′ which produced 360-bp amplification bands. All experiments were performed independently in triplicate. Image analysis and quantification were performed using the SmartView Pro 1200 Imager System (Major Science, USA).

### Cloning of cytochrome P450 monooxygenase

Two different protocols were used to clone the CYP450MO using two vectors: the pJET1.2/blunt cloning vector and pGAPDH-EGFP vector [5]. To confirm mRNA sequencing, the amplified CYP450MO was converted to blunt-ended using Pfu S^+^ DNA polymerase and then ligated with the pJET1.2/blunt cloning vector. The CYP450MO sequence was amplified by PCR using the ATCC_30010 cellular cDNA as the template. To amplify the cDNA encoding CYP450MO, forward CYP450MO _F (5′ – ATG CTG TGG TCG CTG ATT GTT GCG G – 3′) and reverse CYP450MO _R (5′ – GGG CAG TGG TAC GTT TGC GGC AAA – 3′) primers were used. The CYP450MO was cloned into the pJET1.2/blunt cloning vector using a CloneJET PCR Cloning kit (Thermo Fisher Scientific). A CYP450MO fragment was inserted into the pGAPDH-EGFP vector using NdeI/SpeI sites. To amplify the cDNA encoding CYP450MO, forward NdeI_CYP450MO_F (5′ – AAC ATA TGC TGT GGT CGC TGA TTG TTG CGG – 3′) and reverse SpeI_CYP450MO_R (5′ – ACA CTA GTG GGC AGT GGT ACG TTT GCG – 3′) primers were used. All plasmids were transformed to DH5α competent *E. coli* for replication and construction.

### Phylogenetic analysis of AcCYP450MO

We conducted blastp with the peptide sequence of AcCYP450MO against the NCBI nr database (National Center for Biotechnology Information) and retrieved the sequences of the top 100 hits. These sequences were aligned with the “hmmalign” program of the HMMER package v.3.1b2, according to the “cytochrome P450” domain in the pfam database. With the best protein substitution model “JTT + G + I” predicted by MEGA v.7.0 [17], as well as a bootstrap analysis of 100, a maximum likelihood phylogeny was reconstructed with raxml v.8.2.12 [33]. In addition, the functional domain of cytochrome P450 was predicted with the “hmmscan” program of the HMMER package. Structural similarity was assessed by an online tool “Phyre2” [14].

### Cell electroporation of *A. castellanii*

For electroporation, cells were counted using a hemocytometer and centrifuged at 3000 rpm for 3 min to remove the medium. *Acanthamoeba* cells were resuspended in PAS to a final count of 5 × 10^6^ cells/mL and placed in an Eppendorf tube. Ten micrograms of plasmid DNA were added to the Eppendorf tube, followed by PAS to a final volume of 800 μL. The mixture was gently mixed and dispensed into a 4-mm cuvette. Using Gene Pulser Xcell^TM^, the protocol was set as follows: 150 V, 10 ms. After electroporation, the cuvettes containing cells were placed on ice for 10 min, and cells were transferred to a T-75 flask containing PYG for incubation at 28 °C overnight. Stable transformants were selected using 40 μg/mL Geneticin (G418).

### Survival rates of CYP450MO-overexpressing *A. castellanii*

CYP450MO-overexpressing amoeba cells were seeded at a density of 5 × 10^6^ cells/mL in a 6-well plate and treated with 0.01% PHMB for different times, counted using a hemocytometer, and stained using trypan blue.

### Statistical analysis

Data are presented as mean ± standard deviation (SD) from three independent experiments. Student’s *t*-test was used for statistical analysis. Statistical significance was set at *p* < 0.05.

## Results

### The sequencing of cytochrome P450 monooxygenase

**Figure 1:**
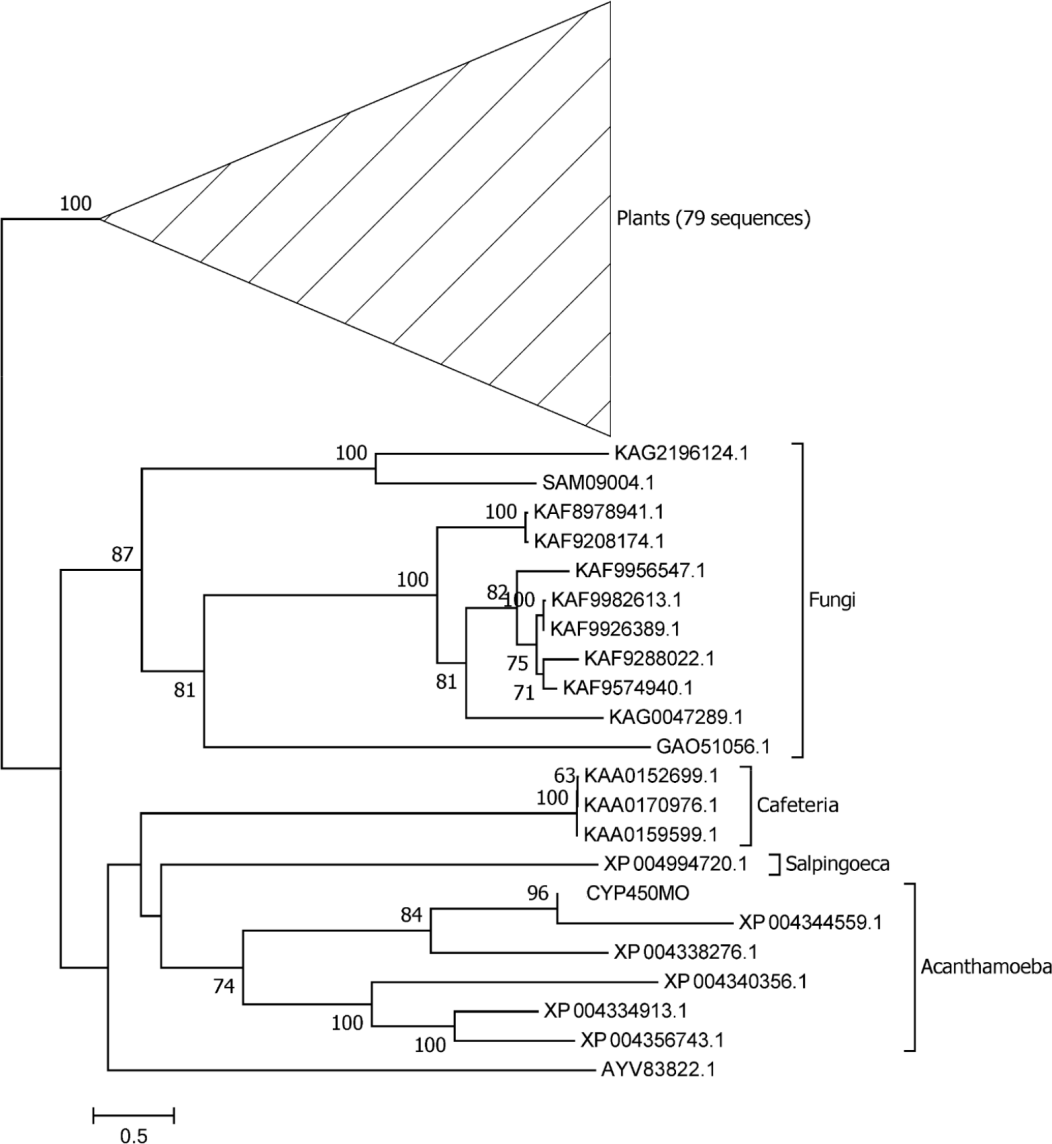
Maximum-likelihood phylogeny of the top 100 peptides closely related to CYP450MO. The numbers next to branches indicate bootstrap support.

**Figure 2:**
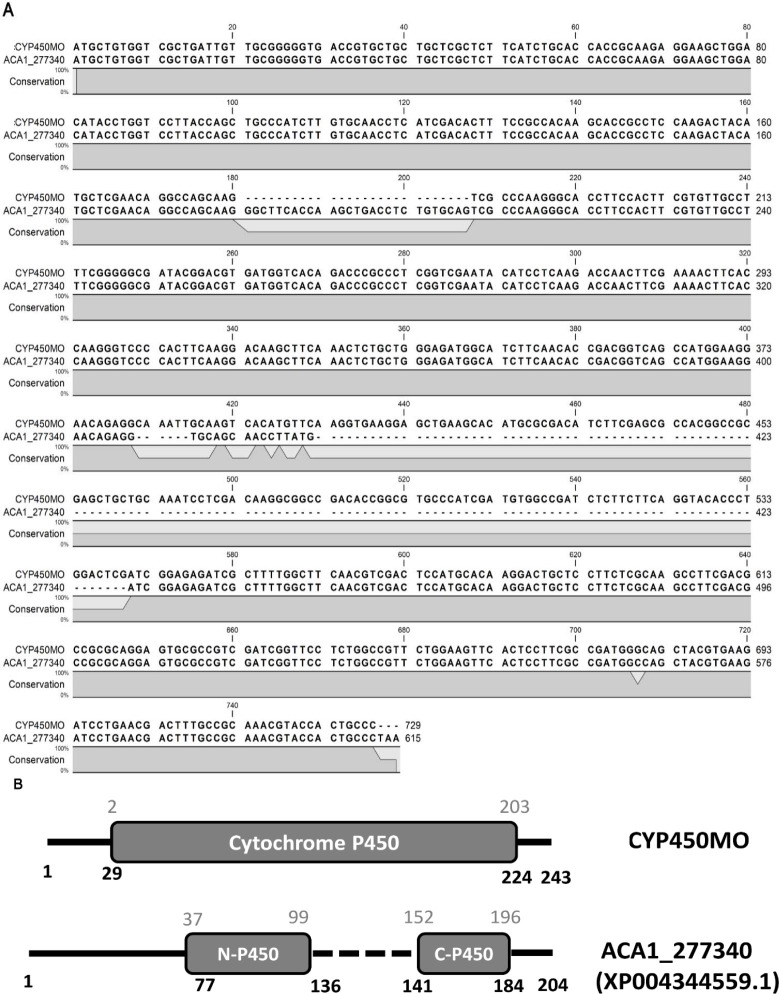
Sequence alignment between CYP450MO and ACA1_277340. (A) Alignment of coding sequences. (B) Schematic representation of the alignment of the cytochrome P450 domain. The numbers in black indicate the position on peptides, while the numbers in grey stand for the position of the hmm model of cytochrome p450 in the pfam annotation database.

CYP450s are widely distributed throughout different organisms ranging from protozoa to mammals [9, 32, 40]. In *Acanthamoeba*, we found 27 CYP450 enzymes (Table 1); moreover, only one CYP450 contained a monooxygenase domain (cytochrome P450 monooxygenase, ACA1_277340) to catalyze a variety of substrates with one oxygen atom [35]. To confirm the mRNA sequence of CYP450MO, we amplified the cDNA using ATCC_30010 cellular cDNA as the template. Compared to the sequences in the NCBI-nr database, we found many differences in the CYP450MO of ATCC_30010 cellular cDNA. We conducted a phylogenetic analysis on CYP450MO and the most similar peptides in GenBank. All peptides of Acanthamoeba formed a monophyletic clade, next to sequences of *Salpingoeca* (a Choanoflagellate) (Fig. 1). In the clade, CYP450MO was closely related to ACA1_277340 (XP004344559.1). When comparing with the coding sequence with ACA1_277340, their 5′ and 3′ ends were identical, while the major difference occurred in the completeness of the cytochrome P450 domain (Fig. 2). CYP450MO possessed a full structure, but the domain was truncated in ACA1_277340 (Fig. 2B). Moreover, phyre2 analysis indicated that CYP450MO showed 99.9% confidence on a high similarity to the structure of human cytochrome P450 2a6. These results indicated that CYP450MO was more likely to show full function than that of ACA1_277340.

**Table 1:** Twenty seven related CYP450 enzymes in *Acanthamoeba castellanii*.

Name	ID	Description
ACA1_290950	ID: 14926367	cytochrome p450 superfamily protein [*Acanthamoeba castellanii str. Neff*]
ACA1_175170	ID: 14925874	cytochrome p450 superfamily protein [*Acanthamoeba castellanii str. Neff*]
ACA1_174810	ID: 14925848	cytochrome p450 superfamily protein [*Acanthamoeba castellanii str. Neff*]
ACA1_254730	ID: 14923340	cytochrome p450 superfamily protein [*Acanthamoeba castellanii str. Neff*]
ACA1_046130	ID: 14922831	cytochrome p450 superfamily protein [*Acanthamoeba castellanii str. Neff*]
ACA1_385730	ID: 14922709	cytochrome p450 superfamily protein [*Acanthamoeba castellanii str. Neff*]
ACA1_183160	ID: 14922274	cytochrome p450 superfamily protein [*Acanthamoeba castellanii str. Neff*]
ACA1_278030	ID: 14921744	cytochrome p450 superfamily protein [*Acanthamoeba castellanii str. Neff*]
ACA1_277340	ID: 14921686	cytochrome P450 monooxygenase, putative [*Acanthamoeba castellanii str. Neff*]
ACA1_054840	ID: 14921608	cytochrome p450 superfamily protein [*Acanthamoeba castellanii str. Neff*]
ACA1_236320	ID: 14919834	cytochrome p450 superfamily protein [*Acanthamoeba castellanii str. Neff*]
ACA1_372100	ID: 14918886	cytochrome p450 superfamily protein [*Acanthamoeba castellanii str. Neff*]
ACA1_065930	ID: 14918208	cytochrome p450 superfamily protein [*Acanthamoeba castellanii str. Neff*]
ACA1_202250	ID: 14916956	cytochrome p450 superfamily protein [*Acanthamoeba castellanii str. Neff*]
ACA1_178260	ID: 14916894	Cytochrome P450, putative [Acanthamoeba *castellanii str. Neff*]
ACA1_019600	ID: 14915577	cytochrome p450 superfamily protein [*Acanthamoeba castellanii str. Neff*]
ACA1_241260	ID: 14913884	cytochrome p450 superfamily protein [*Acanthamoeba castellanii str. Neff*]
ACA1_100440	ID: 14913746	cytochrome p450 superfamily protein [*Acanthamoeba castellanii str. Neff*]
ACA1_095400	ID: 14913279	cytochrome p450 superfamily protein [*Acanthamoeba castellanii str. Neff*]
ACA1_375490	ID: 14912773	cytochrome P450, family 4, subfamily b, polypeptide 1, putative [*Acanthamoeba castellanii str. Neff*]
ACA1_033760	ID: 14912706	cytochrome p450 superfamily protein [*Acanthamoeba castellanii str. Neff*]
ACA1_353190	ID: 14912519	cytochrome p450 superfamily protein [*Acanthamoeba castellanii str. Neff*]
ACA1_338060	ID: 14911366	cytochrome p450 superfamily protein [*Acanthamoeba castellanii str. Neff*]
ACA1_096520	ID: 14913466	cytochrome p450 superfamily protein [*Acanthamoeba castellanii str. Neff*]
ACA1_096500	ID: 14913225	cytochrome p450 superfamily protein [*Acanthamoeba castellanii str. Neff*]
ACA1_374460	ID: 14912855	cytochrome p450 superfamily protein [*Acanthamoeba castellanii str. Neff*]
ACA1_139550	ID: 14914785	nadph cytochrome P450, putative [*Acanthamoeba castellanii str. Neff*]

### The function of CYP450MO in *Acanthamoeba*

**Figure 3:**
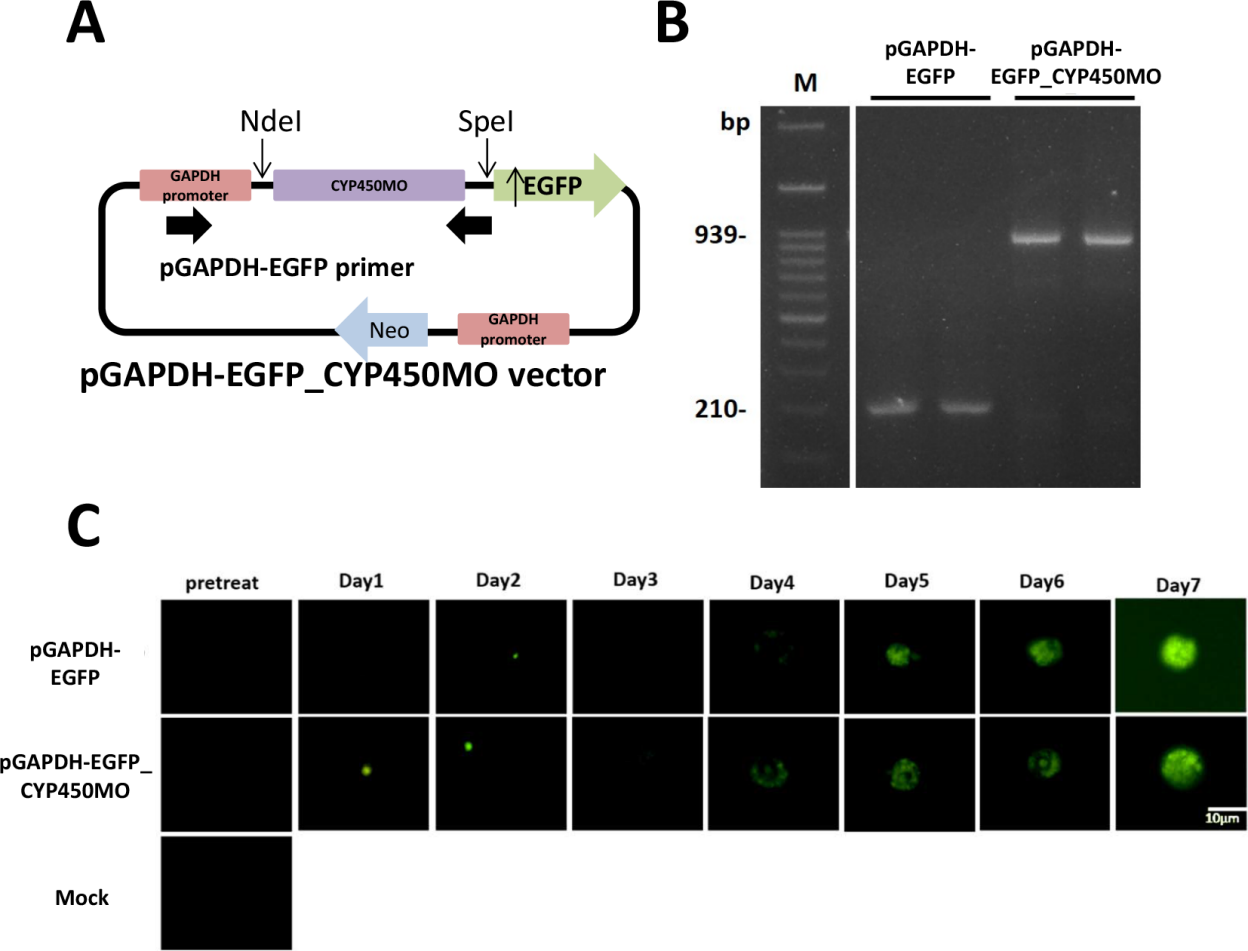
CYP450MO overexpression in *Acanthamoeba* (ATCC_30010). (A) Schematic of the pGAPDH-EGFP-CYP450MO vector. (B) Genomic DNA of *Acanthamoeba* transfected in the pGAPDH-EGFP-CYP450MO vector detected by PCR. (C) *Acanthamoeba* transfected with pGAPDH-EGFP and pGAPDH-EGFP-CYP450MO vector (green) incubated for 7 days and examined using a fluorescence microscope.

To determine whether CYP450MO of *Acanthamoeba* can affect PHMB drug degradation, the enzyme was overexpressed by the pGAPDH-EGFP vector. A CYP450MO fragment was inserted into the pGAPDH-EGFP vector using NdeI/SpeI sites (Fig. 3A). After transfection in *Acanthamoeba* by electroporation for 14 days, the pGAPDH-EGFP-CYP450MO vector was expressed. To confirm that the pGAPDH-EGFP-CYP450MO vector was transfected into *Acanthamoeba*, the DNA extracted from *Acanthamoeba* was amplified using the pGAPDH-EGFP primers (Fig. 3B). The EGFP-CYP450MO fusion protein was also expressed in *Acanthamoeba* using a Cell^R^ microscope (Olympus America, Inc., USA) for 7 days (Fig. 3C).

**Figure 4:**
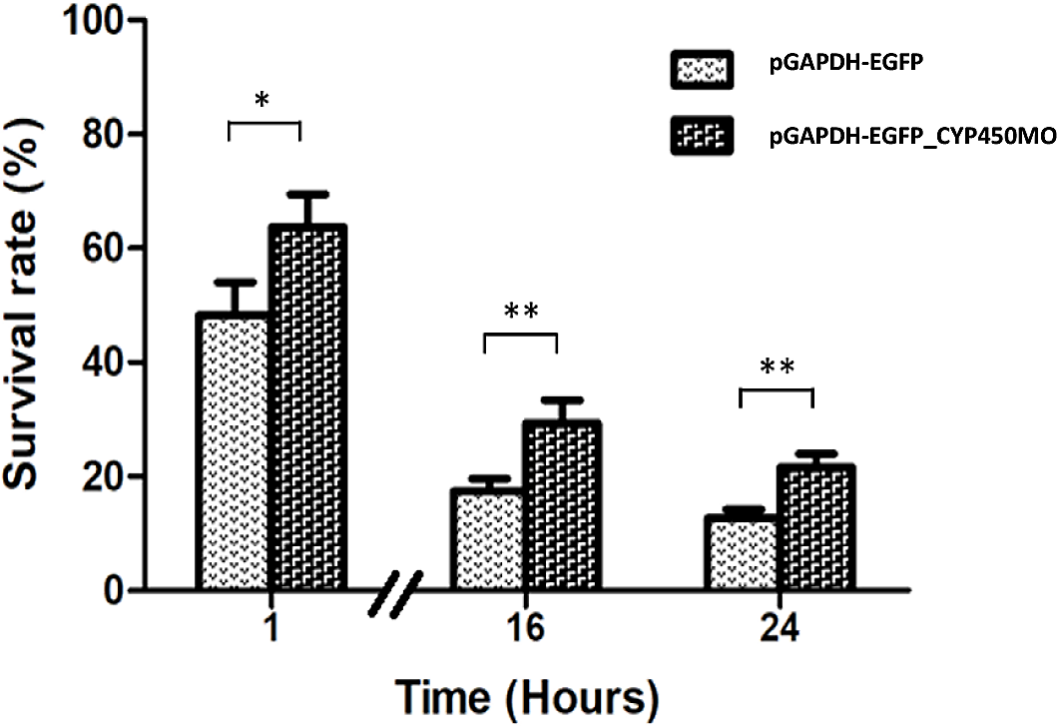
Survival rate of *Acanthamoeba* treated with PHMB. Survival rate of *Acanthamoeba* cells transfected with pGAPDH-EGFP and pGAPDH-EGFP-CYP450MO vector incubated with 0.01% PHMB for 1, 16, and 24 h. Data are presented as mean ± standard deviation (SD).

*Acanthamoeba-*transfected pGAPDH-EGFP-CYP450MO vectors were treated with 0.01% PHMB. The results showed that the survival rates of *Acanthamoeba-*transfected pGAPDH-EGFP-CYP450MO vector were higher than those of the control at 1, 16, and 24 h (Fig. 4). Hence, we suggest that *Acanthamoeba* overexpressing CYP450MO may be resistant to PHMB drug, enhancing survival rates.

### CYP450MO and encystation in *Acanthamoeba*

**Figure 5:**
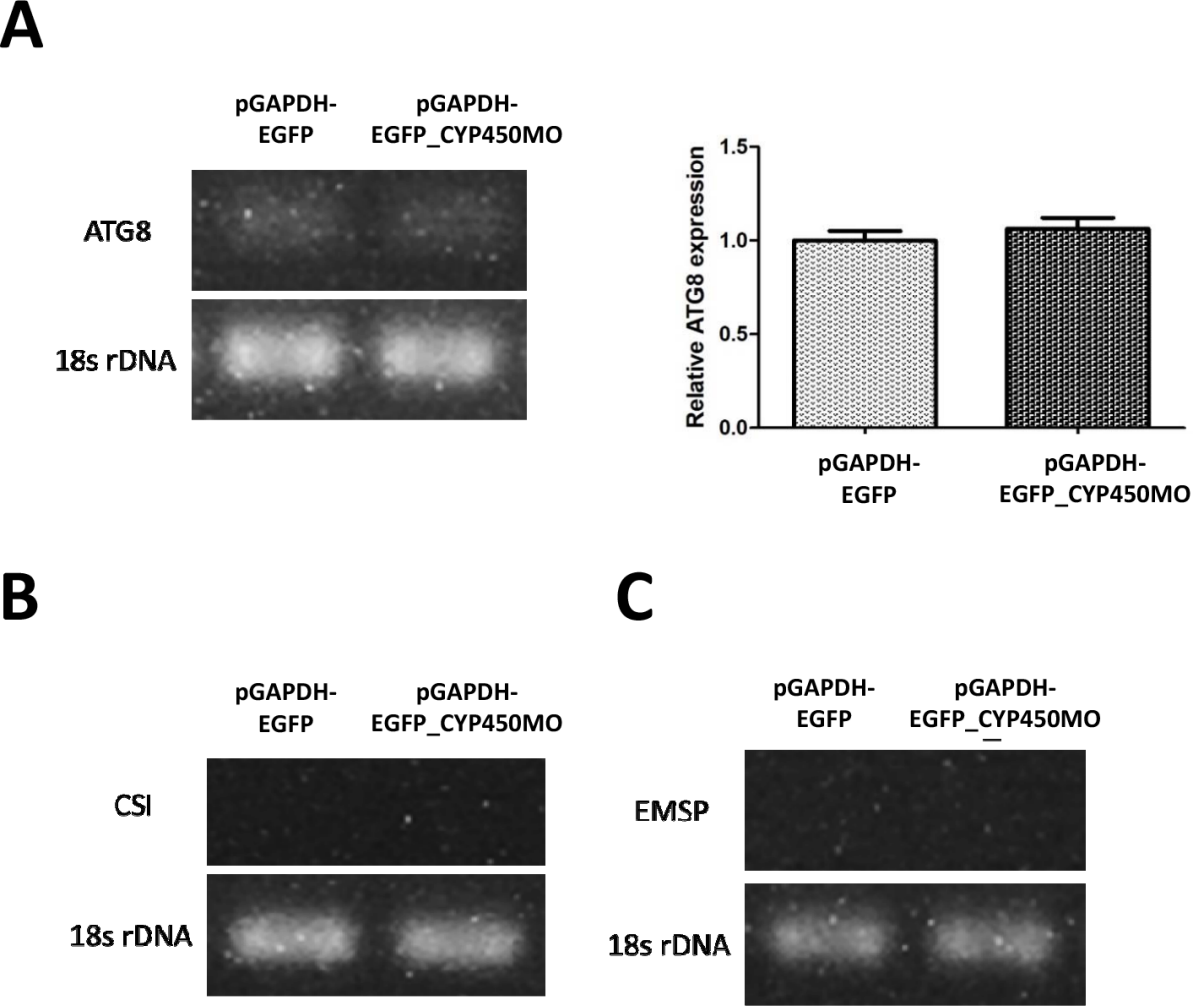
mRNA expression of encystation genes in *Acanthamoeba* transfected with pGAPDH-EGFP and pGAPDH-EGFP-CYP450MO vector. mRNA expression of ATG8 (A), CSI (B), and EMSP (C). 18s rDNA expression was used as the control (*p ≤ 0.05).

A previous study showed that clinical isolates can resist drugs by encystation to avoid environmental stress [10]. To determine whether *Acanthamoeba-*transfected pGAPDH-EGFP-CYP450MO vector induced encystations to avoid PHMB drug lysis, gene-related encystations were detected. CSI, EMSP and ATG8 identified in *Acanthamoeba* are involved in the encystation mechanism [16, 27]. The results showed that ATG8 expression was not significantly different between *Acanthamoeba-*transfected pGAPDH-EGFP and pGAPDH-EGFP-CYP450MO (Fig. 5A). CSI and EMSP expression levels were also not significantly different between *Acanthamoeba-*transfected pGAPDH-EGFP and pGAPDH-EGFP-CYP450MO (Figs. 5B and 5C). Hence, we suggest that *Acanthamoeba-*transfected pGAPDH-EGFP-CYP450MO may not induce encystation to resist PHMB drug lysis.

## Discussion

*Acanthamoeba castellanii* has 27 CYP450 genes compared to the 57 CYP450 genes in the human genome [29]. The CYP450 genes related to drug metabolism in humans are CYP2C9, CYP2C19, CYP2D6, and CYP3A4 [11]. In nematodes, *Caenorhabditis elegans* encodes 80 CYP450 genes. Some CYPs in *C. elegans* such as *cyp35a2, cyp35a5,* and *cyp35c1* play a role in albendazole (ABZ), an anti-helminthic medication [8, 18]. However, in protozoa such as *Toxoplasma gondii*, the CYP450 gene exists as a single copy. The CYP450 of *T. gondii* plays an important role in developing resistance to drugs such as quinine, mefloquine, and clarithromycin [40]. In this study, we found 27 related CYP450 enzymes in *A. castellanii* (Table 1). A previous study showed that CYP450 genes in humans were observed to enhance gene diversity by alternative RNA splicing [34]. Therefore, it is likely that CYP450s are produced from the *Acanthamoeba* gene by alternative splicing to metabolize different drugs.

In this study, CYP450MO induced PHMB drug metabolism for the survival of *Acanthamoeba*, as CYP450MO overexpression enhanced the resistance of *Acanthamoeba*. Moreover, in previous studies, strains resistant to encystation were also transformed into pseudocysts or cysts under the effects of PHMB drug stress [10, 23]. ATG8 in *Acanthamoeba* encystation plays an important role in autophagy against drug therapy [12]. CSI and EMSP have also been identified in *Acanthamoeba* and are involved in the encystation mechanism [16, 27]. However, ATG8, CSI, and EMSP levels were not significantly different between *Acanthamoeba-*transfected pGAPDH-EGFP and pGAPDH-EGFP-CYP450MO (Fig. 5). Hence, we suggest that *Acanthamoeba* may not express encystation-related genes against PHMB drug lysis.

CYP450s are known to catalyze a variety of chemical reactions and attack substrates from electron transfer chains. On the electron transfer chains, CYP450s incorporate oxygen atoms into the substrate molecule by transferring electrons from NAD(P)H [31]. Monooxygenase systems depend on monooxygenase activity catalyzing one oxygen atom in the substrate molecule. Many drug metabolic processes catalyzed by monooxygenase involve the oxidation of endogenous and exogenous substrates [35]. In this study, we also found that the survival rates of *Acanthamoeba-*transfected pGAPDH-EGFP-CYP450MO vector were higher than those of the control after PHMB treatment (Fig. 4). Hence, we suggest that CYP450MO in *Acanthamoeba* may catalyze PHMB drug metabolism to exogenous substrates and be secreted into the extracellular environment. In the future, we aim to focus on CYP450MO as a drug target to potentially treat AK.

## Conclusions

In this study, we overexpressed CYP450MO in *Acanthamoeba* to investigate PHMB drug resistance. *Acanthamoeba* with CYP450MO-overexpression had higher survival rates than those of the control cells after PHMB treatment. We suggest that CYP450MO in *Acanthamoeba* may catalyze PHMB drug metabolism to enhance survival rates after PHMB treatment.

## Availability of data and materials

Data supporting the conclusions of this article are included within the article. The datasets used and/or analyzed during the present study are available from the corresponding author upon reasonable request.

## Competing interests

All authors declare that they have no conflicts of interest.

## Funding

This research was supported by the Ministry of Science and Technology (MOST) through funding to WCL (grant MOST 110-2628-B-006-32) and by the National Cheng Kung University Hospital through funding to FCH.
